# Thrombomodulin Contributes to Gamma Tocotrienol-Mediated Lethality Protection and Hematopoietic Cell Recovery in Irradiated Mice

**DOI:** 10.1371/journal.pone.0122511

**Published:** 2015-04-10

**Authors:** Rupak Pathak, Lijian Shao, Sanchita P. Ghosh, Daohong Zhou, Marjan Boerma, Hartmut Weiler, Martin Hauer-Jensen

**Affiliations:** 1 Division of Radiation Health, Department of Pharmaceutical Sciences, College of Pharmacy, University of Arkansas for Medical Sciences, Little Rock, AR, United States of America; 2 Armed Forces Radiobiology Research Institute, USUHS, Bethesda, MD, United States of America; 3 Blood Research Institute, Blood Center of Wisconsin and the Medical College of Wisconsin, Milwaukee, WI, United States of America; 4 Surgical Service, Central Arkansas Veterans Healthcare System, Little Rock, AR, United States of America; Lunenfeld-Tanenbaum Research Institute, CANADA

## Abstract

Systemic administration of recombinant thrombomodulin (TM) confers radiation protection partly by accelerating hematopoietic recovery. The uniquely potent radioprotector gamma tocotrienol (GT3), in addition to being a strong antioxidant, inhibits the enzyme hydroxy-methyl-glutaryl-coenzyme A reductase (HMGCR) and thereby likely modulates the expression of TM. We hypothesized that the mechanism underlying the exceptional radioprotective properties of GT3 partly depends on the presence of endothelial TM.

*In vitro* studies confirmed that ionizing radiation suppresses endothelial TM (about 40% at 4 hr after 5 Gy γ-irradiation) and that GT3 induces TM expression (about 2 fold at the mRNA level after 5 μM GT3 treatment for 4 hr). *In vivo* survival studies showed that GT3 was significantly more effective as a radioprotector in TM wild type (*TM^+/+^*) mice than in mice with low TM function (*TM^Pro/-^*). After exposure to 9 Gy TBI, GT3 pre-treatment conferred 85% survival in *TM^+/+^* mice compared to only 50% in *TM^Pro/-^*. Thus, GT3-mediated radiation lethality protection is partly dependent on endothelial TM. Significant post-TBI recovery of hematopoietic cells, particularly leukocytes, was observed in *TM^+/+^* mice (p = 0.003), but not in *TM^Pro/-^* mice, despite the fact that GT3 induced higher levels of granulocyte colony stimulating factor (G-CSF) in *TM^Pro/-^* mice (p = 0.0001). These data demonstrate a critical, G-CSF-independent, role for endothelial TM in GT3-mediated lethality protection and hematopoietic recovery after exposure to TBI and may point to new strategies to enhance the efficacy of current medical countermeasures in radiological/nuclear emergencies.

## Introduction

The endothelial glycoprotein, thrombomodulin (TM), has important anti-coagulant, anti-inflammatory, and cytoprotective properties and appears to regulate normal tissue radiation responses [1, 2]. Hence, microvascular TM decreases after exposure to ionizing radiation [3], and treatment with exogenous recombinant TM significantly improves post-TBI survival in mice, partly by accelerating hematopoietic cell recovery [4].

Analogs of vitamin E (tocols) are currently being developed as radioprotective agents. Among the tocols, gamma-tocotrienol (GT3), an unsaturated analogue of tocopherol, has particularly potent radioprotective properties compared with other tocols [5, 6]. However, while GT3 is the natural compound with the largest dose reduction factor discovered to date, the mechanisms underlying the superior radioprotective efficacy of GT3 are not completely understood.

Tocols exert their biological effects primarily by virtue of their antioxidant properties and by their ability to inhibit the enzyme hydroxyl-methyl-glutaryl-coenzyme A reductase (HMGCR), similar to the lipid lowering drugs statins. Comparison of the antioxidant activities of tocols suggest that the superior radioprotective efficacy of GT3 is not likely because of differences in antioxidant properties [7], but rather due to the remarkably strong inhibitory effect of GT3 on HMGCR compared to other tocols [8]. We and others have demonstrated that inhibition of HMGCR protects lung and vascular endothelium from radiation injury *in vivo* [6, 9], thus pointing to this particular property of GT3 as a possible basis of its unique efficacy as a radioprotector.

HMGCR inhibitors prominently upregulate TM via mechanisms involving Kruppel-like transcription factors and nitric oxide-induced dissociation of heat shock factor 1 from heat shock protein 90 [10]. In fact, this effect is so prominent that, after the first description of the effect of HMGCR inhibitors on TM [11], TM is commonly used as a read-out when assessing the effect of HMGCR inhibitors *in vitro*. We hypothesized that the radioprotective superiority of GT3 over other tocols may be related specifically to its ability to upregulate endothelial TM.

Our findings confirm that GT3 increases endothelial cell TM *in vitro* and that the efficacy of GT3 as a radioprotective agent *in vivo* to a significant degree depends on the presence of endothelial TM. Surprisingly, however, the levels of granulocyte colony stimulating factor (G-CSF) appeared to be unrelated to hematopoietic recovery or to lethality protection, suggesting that this growth factor does not mediate the TM-dependent effect of GT3. Our results corroborate data using recombinant TM or recombinant activated protein C (APC) [4] and suggest that synergistic or additive radioprotection can be achieved by using GT3 and other HMGCR inhibitors in combination with TM and/or APC.

## Materials and Methods

### Cell Lines and Reagents

Primary human umbilical vein endothelial cells (HUVECs) and immortal human endothelial cells (EA.hy926) were obtained from American Type Culture Collection (ATCC, Manassas, VA). The EGM-2 Bullet kit and HEPES-buffered saline solution, to culture HUVEC cells, were from Lonza (Walkersville, MD). EA.hy926 cell were maintained in Dulbecco’s Modified Eagle Medium (DMEM). Heat-inactivated Fetal Bovine Serum (FBS) was obtained from Atlanta Biologicals (Lawrenceville, GA). To dislodge adherent cells 0.05% trypsin was obtained from HyClone (Pittsburgh, PA). RIPA cell lysis buffer, Tris-Glysine SDS gel running buffer, transfer buffer, and laemmli SDS 4X sample buffer were procured from Boston BioProducts (Ashland, MA). Recombinant Hirudin from yeast was obtained from American Diagnostica Inc. (Stamford, CT). Chromogenix S-2366 was obtained from DiaPharma Group Inc. (West Chester, OH). Primary TM antibody was procured from Novus Biologicals (Catalogue number: NBP1-95319; Littleton, CO) and goat anti-rabbit IgG-HRP conjugated secondary antibodies were obtained from Santa Cruz Biotechnology, Inc. (Catalogue number: SC-2004; Santa Cruz, CA). TE buffer and DPBS were obtained from Invitrogen (Grand Island, NY). GT3 was procured from Yasoo Health Inc. (Johnson City, TN).

### Irradiation

Un-anesthetized mice were exposed to a single whole-body radiation dose in a Shepherd Mark I, model 25 ^137^Cs irradiator (J. L. Shepherd & Associates, San Fernando, CA). During irradiation, the mice were placed in a well-ventilated chamber made specifically for irradiation of mice (J.L. Shepherd & Associates). The chamber is made of aluminum with a well-ventilated Plexiglas lid, divided into eight equal ‘‘pie slice” compartments by dividers made of T-6061 aluminum with a gold anodized coating. The average dose rate was 1.12 Gy per min and was corrected for decay each day. All radiation experiments were performed in the morning to minimize possible diurnal effects.

### Ethics Statement

All animal studies were carried out in strict accordance with the recommendations in the Guide for the Care and Use of Laboratory Animals of the National Institutes of Health. The animal protocol was approved by the Institutional Animal Care and Use Committee of the University of Arkansas for Medical Sciences. All animals were housed under standard air-conditioned animal facility at 20 ± 2°C with 10–15 hourly cycles of fresh air and free access to standard rodent food and water. Upon arrival, the mice were held in quarantine for 1 week and provided certified rodent chow.

### Animals

TM wild-type (*TM* ^*+/+*^) and TM mutant (*TM* ^*Pro/-*^) male and female mice were used for the study. Animals were housed in conventional cages under standardized conditions with controlled temperature and humidity and a 12–12-h day-night light cycle. Animals had free access to water and chow (Harlan Teklad laboratory diet 7012, Purina Mills, St. Louis, MO). Twenty-four hours before irradiation, mice received a single dose of GT3 (400 mg/kg) or the excipient alone by subcutaneous injection.

The homozygous TM-deficient genotype is embryonically lethal. Therefore, mice with very low TM activity were generated by combining two different transgenic mouse strains on C57BL/6 background, haploinsufficient heterozygous TM-deficient (*TM* ^*+/-*^) mice, and *TM* ^*Pro/Pro*^ mice where a glutamic acid residue has been replaced with a proline residue, thus reducing the ability to generate activated protein C by 80% [12]. By crossing *TM* ^*+/-*^ with *TM* ^*Pro/Pro*^ mice, *TM* ^*Pro/-*^ with <5% of normal TM activity were generated. Wild-type control mice (*TM* ^*+/+*^) were obtained from crosses of *TM* ^*+/-*^ with C57BL/6 mice.

### Survival Studies

For studies of post-irradiation survival, a total of 46 mice were used, in which 25 wild type (*TM* ^*+/+*^) and 21 thrombomodulin mutant mice (*TM* ^*Pro/-*^) mice were exposed to 9 Gy of total body irradiation (TBI). The dose of 9 Gy TBI is known to cause severe hematopoietic and gastrointestinal injury resulting in radiation lethality in more than 80% of CD2F1 mice. Before 24 hr of radiation exposure *TM* ^*+/+*^ mice were injected subcutaneously (s.c) either with vehicle (5% Tween-80 in normal saline, n = 12; male = 5 and female = 7) or 400 mg/kg body weight GT3 (n = 13; male = 6 and female = 7). *TM* ^*Pro/-*^ mice were treated in the same manner either with vehicle (n = 11; male = 4 and female = 7) or 400 mg/kg body weight of GT3 (n = 10; male = 4 and female = 6). The GT3 dose was selected based on our previously published GT3 efficacy study in radiation lethality protection[13]. Mice were monitored up to 30 days after TBI, and the numbers of dead or moribund mice were recorded twice daily. Animals that were moribund (more than 25% weight loss, lethargy, huddling and/or shivering, loss of appetite, hunched posture, severe diarrhea, and vocalization) were euthanized without delay by gradual exposure to CO_2_ in a chamber until all respiratory effort has ceased and no detectable heart beat or eye ball movement are observed. In this study, a total of 26 mice were found dead out of 46 animals. We euthanized 9 moribund animals. The remaining 17 non-moribund mice (out of 26 dead animals) died during the course of radiation lethality study as a direct result of radiation insult, without showing any of the overt clinical signs used for humane endpoints and were found dead during the daily monitoring. All survived animals (20 out of 46 animals) were humanely euthanized after 30 day of irradiation. Kaplan-Meier survival curves were plotted and analyzed with the log-rank test.

### RNA Extraction, cDNA Preparation, and Quantitative Real-time PCR (qRT-PCR)

HUVEC and EA.hy926 cells were washed twice with calcium- and magnesium-free cold PBS. Total RNA was extracted using the RNeasy Plus Mini Kit from Qiagen (Valencia, CA), per the manufacturer’s instructions. Immortalized EA.hy926 cells between passage numbers 25 and 30 and HUVEC cells between passage numbers 3 and 7 were used for RNA extraction. Each RNA sample was subsequently treated with TURBO Dnase (Ambion, Grand Island, NY) to remove traces of genomic DNA. RNA quality was assessed and quantity was measured for each sample by using the Agilent 2100 Bioanalyzer (Agilent Technologies, Santa Clara CA). Samples with an RNA integration number (RIN) of 10 were used for cDNA preparation. A total of 2 μg RNA was used to prepare cDNA using a high-capacity cDNA Reverse Transcription kit from Applied Biosystems (Carlsbad, CA). qRT-PCR for different genes of interest was carried out using an ABI Prism 7000 Sequence Detection System (Applied Biosystems). Standard real-time PCR (50°C for 2 minutes and 95°C for 10 minutes, followed by 50 cycles of 95°C for 15 seconds and 60°C for 60 seconds) with the TaqMan 2X Universal PCR master mix from Applied Biosystems was used to quantitatively measure mRNA. Gene expression assays were performed for TM (Hs00264920_s1) and 18s (Hs03928990_g1) using the inventoried TaqMan assay from Applied Biosystems with FAM-labeled probes. Expression of each gene of interest was normalized to 18s ribosomal RNA for each sample. Relative mRNA expression was calculated using the comparative C_T_ (2^-ΔΔCt^) method.

### Western Blot Analysis

Endothelial cells were lysed with RIPA buffer containing the cocktail of protease and phosphatase inhibitors, and samples were subsequently subjected to electrophoresis on NuPAGE Novex 4–12% Bis-Tris Gel (Invitrogen) at 100 V constant current for 2 h after protein quantitation with the BCA Protein Assay Kit (Thermo Scientific, Rockford, IL). EA.hy926 cells between passage numbers 25 and 30 were used for Western blot analysis. Protein samples were transferred onto PVDF membranes (Invitrogen) at 100 V for 1–3 h depending on protein size. Each membrane was blocked with 5% non-fat milk from Thermo Scientific (Rockford, IL) in 1x Tris-Buffered Saline Tween-20 (TBST) for 1 h at room temperature on a shaker. Desired antigens were analyzed using designated antibodies. Primary antibody specific for human TM antigen was added in 5% non-fat milk in 1x TBST at a dilution of 1:1000, and each membrane was incubated overnight with primary antibody at 4°C. Each membrane was washed three times with 1x TBST for 5 min, and then HRP-conjugated secondary antibody was added at a dilution of 1:10000 for 1 h at room temperature. After five washes with 1x TBST, bound antibody was detected using SuperSignal West Pico Chemiluminescent Substrate (Thermo Scientific Pierce, Rockford, IL). Beta-actin was used as the internal control. The blots were then exposed to Kodak XAR-5 films to obtain the fluorographic images.

### Protein C Activation Assay

Cells were seeded at a density of 2 x 10^4^ cells/well in 96-well plates 24 h prior to treatment. Cells were exposed to 5 Gy and incubated for 48 h. Cells were then washed twice with PBS and incubated with a master mix of thrombin (1 nmol/L), protein C (0.5 μmol/L), and buffer in a total volume of 60 μL for 1 h. After 1 h of incubation at 37°C, 20 μL of hirudin (0.2 unit/μL) was added to neutralize thrombin. After ten minutes, 100 μL chromogenic substrate S-2366 (0.5 mM) was added to each well, and the change in absorbance at 405 nm (milli-optical density/min) was measured using a microplate reader (BioTek, Winooski, VT). A standard curve generated with known concentrations of APC was used to determine the concentrations of APC generated in the reaction mixture.

### Blood Cell Counts

At day 30 after 6 Gy of TBI, blood was collected in an EDTA-coated tube by retro-orbital puncture. A sub-lethal dose of radiation (6 Gy) was selected for this study to ensure all the mice survive for the indicated period of time and, at the same time, cause significant depletion in blood cell counts. Peripheral blood cell counts were obtained using a veterinary Hemavet system (Drew Scientific, Dallas, TX) according to the manufacturer’s instructions. For this study a different set of *TM* ^*+/+*^ and *TM* ^*Pro/-*^ mice were used.

### G-CSF ELISA Assay

Concentrations of G-CSF in blood plasma samples were assessed by using a commercially available mouse specific enzyme-linked immunosorbent assay kit (RayBiotech, Inc. Norcross GA), according to the manufacturer’s instructions. Single use plasma aliquots were thawed on ice, diluted 1:100, and measured in duplicate with a plate reader (SpectraMax M5, Molecular Devices) set at an absorbance of 450 nm.

### Cytokine Estimation in Mouse Blood Plasma

A single dose of GT3 (400 mg/kg) was administered sc to *TM* ^*+/+*^ and *TM* ^*Pro/-*^ mice 24 h before retro-orbital blood collection. Cytokines level were measured in plasma samples of *TM* ^*+/+*^ and *TM* ^*Pro/-*^ mice using a Multiplex kit from Millipore (Billerica, MA), according to the manufacturer's instructions.

### Statistical Analysis

The log-rank test was used to compare survival curves. Fisher’s exact test was used to compare survival rates at the end of 30 d, with a Bonferroni correction used to control for type-I error whenever multiple comparisons were used. Mean values with standard errors (SE, when applicable) were reported. Analysis of variance (ANOVA) was used to detect whether there were significant differences between groups. If significant, a Tukey’s post hoc test was used to determine significant differences between particular groups. All statistical tests were two-sided with a 5% significance level. Statistical analysis was performed using Prism (GraphPad Software, La Jolla, CA).

## Results

### Effects of Radiation on Endothelial TM Expression

**Fig 1:**
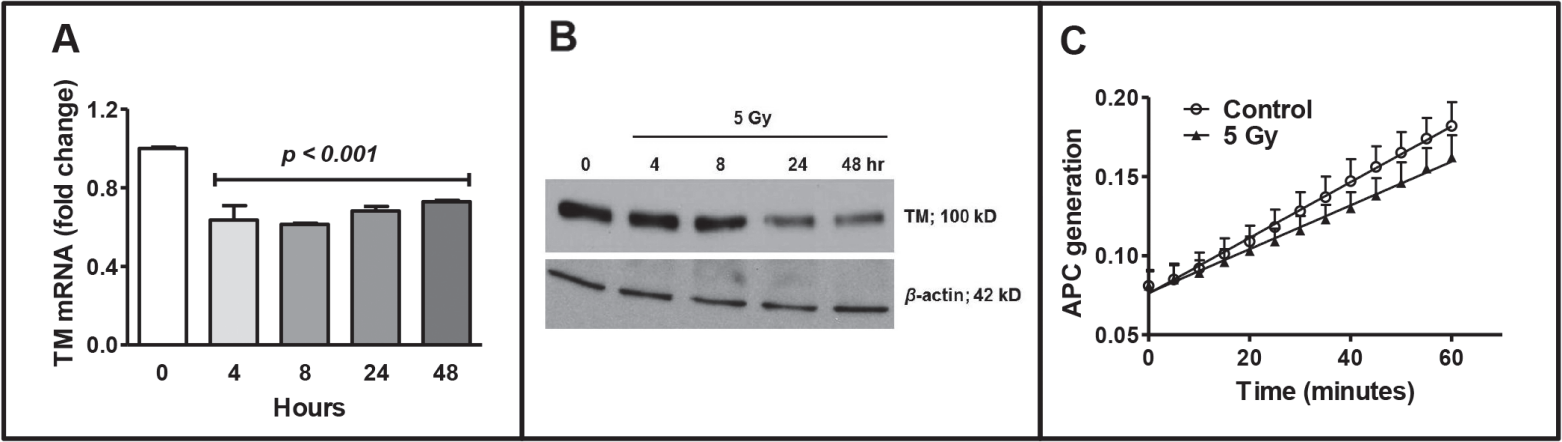
Effect of ionizing radiation on endothelial TM expression. Panel A: Relative TM mRNA fold change in human immortalized endothelial cells, EA.hy926 cells, at different time points after exposure to 5 Gy γ -irradiation. Mean TM mRNA expression in irradiated EA.hy926 cells was significantly lower between 4 and 48 hr as compared to 0 hr. Panel B: Change in TM protein level in EA.hy926 cells after exposure to 5 Gy at indicated post-irradiation times. Panel C: Generation of activated protein C (APC), which indicates the functional activity of TM, in EA.hy926 cells at 48 h after exposure to 5 Gy. The increase in APC generation in irradiated EA.hy926 cells was significantly lower than that in un-irradiated cells. Means and error bars were calculated from at least three independent experiments.

To determine the effect of ionizing radiation on endothelial TM expression, human endothelial cells were exposed to 5 Gy of γ-rays and TM expression was measured at different post-irradiation time intervals at the mRNA and protein levels. The effect of radiation on TM function was also determined by measuring APC generation in irradiated endothelial cells. Significant decrease in TM mRNA (Fig 1A) as well as in TM protein (Fig 1B) expression was observed in endothelial cells at various post-irradiation time points. Increase in APC generation in irradiated cells was significantly less than that in un-irradiated cells (Fig 1C). These data confirm that ionizing radiation causes a decrease in TM expression and function.

### Effect of GT3 on Endothelial TM Expression

**Fig 2:**
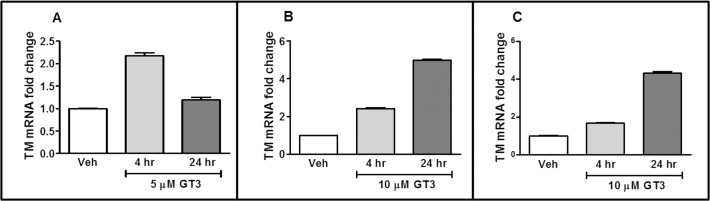
Effect of GT3 on TM expression in human endothelial cells. Relative TM mRNA fold change in human immortalized EA.hy926 endothelial cells (A and B) and human primary endothelial cells, HUVEC cells (C), after treatment with 5 to 10 μM of GT3 or vehicle (veh) at different time intervals as detected by qRT-PCR. GT3 treatment significantly increased (*p<0*.*0001*) TM expression at the mRNA level in both the EA.hy926 cells and HUVECs. Means and error bars were calculated from at least three independent experiments.

To assess the effect of GT3 on endothelial TM expression, human immortalized (Fig 2A and 2B) and primary endothelial cells (Fig 2C) were treated with 5 or 10 μM of GT3 for various times and TM expression at the mRNA level was measured by qRT-PCR. GT3 treatment significantly induced TM mRNA expression in human endothelial cells in a time-dependent manner.

### Effect of GT3 Pre-treatment on Radiation Lethality Protection in TM Wild-type (*TM* ^*+/+*^) and TM Mutant (*TM* ^*Pro/-*^) Mice

**Fig 3:**
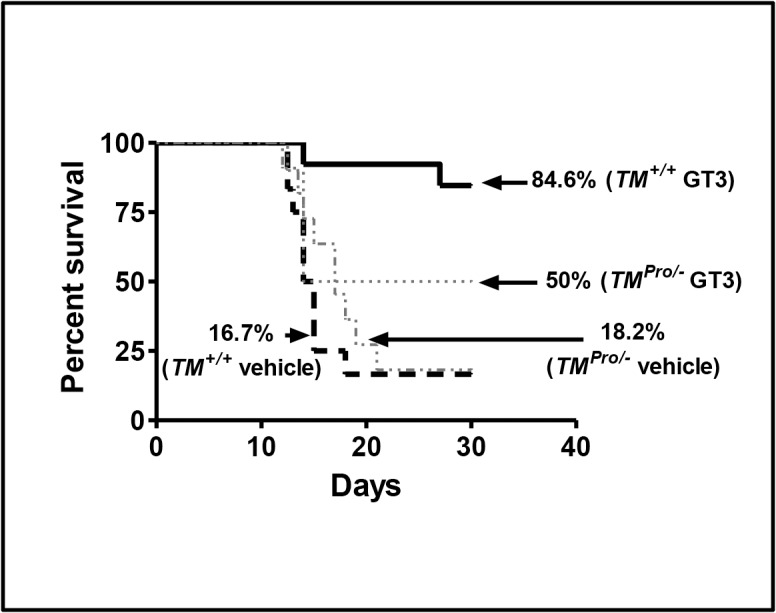
Lethality protection by GT3 in in *TM* ^*+/+*^ and *TM* ^*Pro/-*^ mice after exposure to 9 Gy TBI. *TM* ^*+/+*^ mice were injected subcutaneously (s.c) either with vehicle (5% Tween-80 in normal saline, n = 12; male = 5 and female = 7) or 400 mg/kg body weight GT3 (n = 13; male = 6 and female = 7). *TM* ^*Pro/-*^ mice were treated in the same manner either with vehicle (n = 11; male = 4 and female = 7) or 400 mg/kg body weight of GT3 (n = 10; male = 4 and female = 6). Twenty-four hr later, the mice were exposed to 9 Gy TBI and survival was monitored for 30 days. Log-rank (Mantel-Cox) test revealed significant (*p = 0*.*0003*) lethality protection after GT3 treatment in *TM* ^*+/+*^ mice compared to vehicle treated *TM* ^*+/+*^ controls. In contrast, GT3 treatment did not provide significant (*p = 0*.*29*) lethality protection to *TM* ^*Pro/-*^ mice compared to vehicle treated *TM* ^*Pro/-*^ controls.

Because our *in vitro* studies suggested that GT3 enhances endothelial TM expression, we next investigated the role of TM in GT3-mediated protection against radiation-induced lethality in *TM* ^*+/+*^ and *TM* ^*Pro/-*^ mice. *TM* ^*+/+*^ and *TM* ^*Pro/-*^ mice of both sexes were exposed together to 9 Gy of TBI using a ^137^Cs-irradiatior. Significant lethality protection (*p = 0*.*0003*) was observed in GT3-treated *TM* ^*+/+*^ mice (84.6% survival) compared to vehicle-treated *TM* ^*+/+*^ mice (16.7% survival). Although GT3 pre-treatment also conferred lethality protection in *TM* ^*Pro/-*^ mice (50% survival) compared to vehicle-treated *TM* ^*Pro/-*^ mice (18% survival), this difference did not reach statistical significance (*p = 0*.*29*). Overall, the 30-day radiation survival study revealed that GT3 was only about half as efficacious in *TM* ^*Pro/-*^ compared to *TM* ^*+/+*^ mice (Fig 3). These data clearly suggest that TM plays a critical role in GT3-dependent radiation-induced lethality protection in mice. There was no gender-dependent difference in radiation sensitivity between *TM* ^*+/+*^ and *TM* ^*Pro/-*^ mice.

### Effect of GT3 in Post-TBI Recovery of Hematopoietic Cells

**Fig 4:**
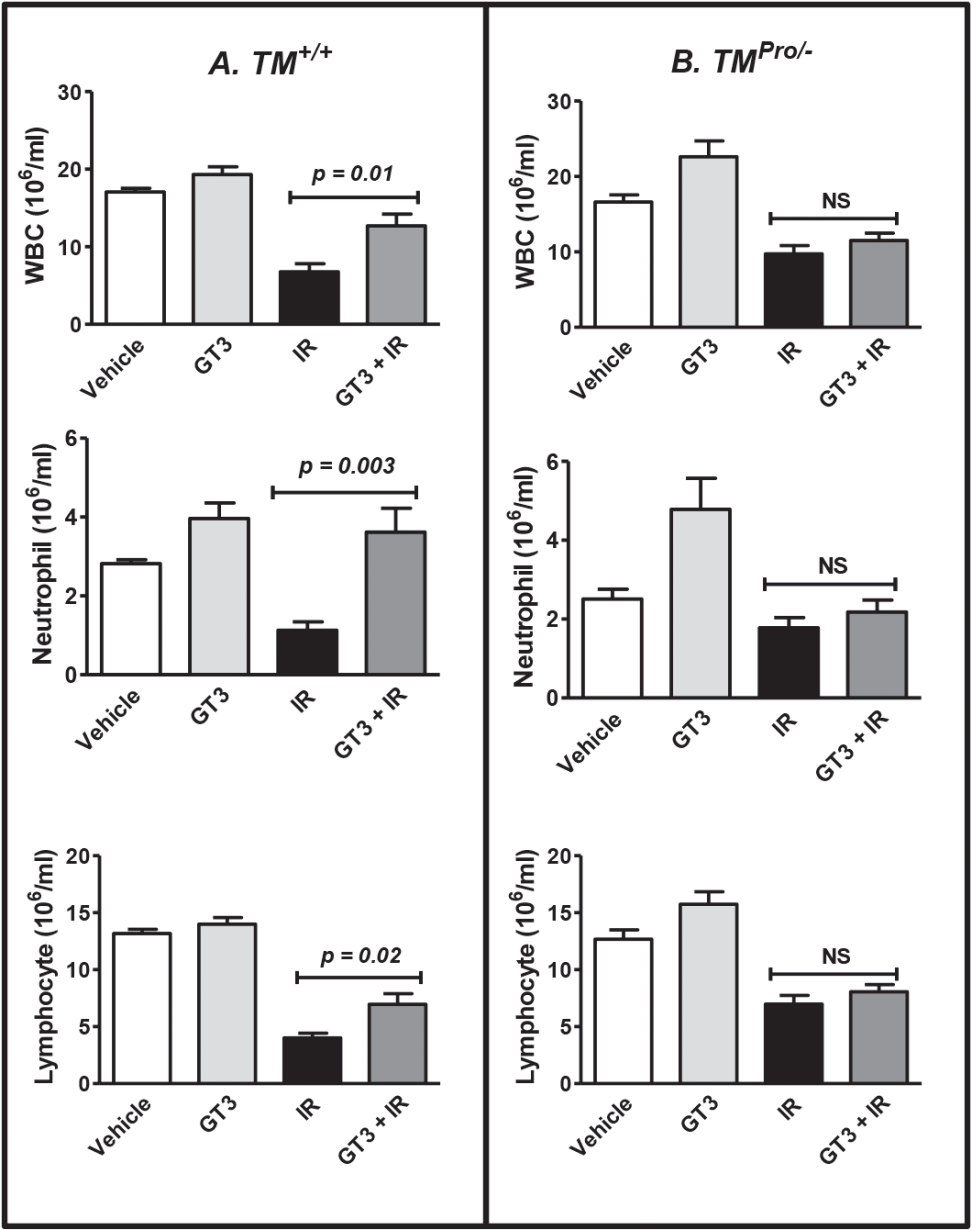
Leucocyte recovery in *TM* ^*+/+*^ and *TM* ^*Pro/-*^ mice pre-treated with GT3 before exposure to 6 Gy TBI. Panel A: A single subcutaneous administration of GT3 (400 mg/kg body weight) 24 h before exposure to 6 Gy of TBI significantly improved total white blood cell (WBC) counts, neutrophil counts and lymphocyte counts in *TM* ^*+/+*^ mice in comparison to their vehicle (5% Tween-80 in normal saline) treated control group on day 30. Similar treatment was unable to improve WBC, neutrophil and lymphocyte counts in *TM* ^*Pro/-*^ mice (Panel B). Four to 5 mice were used except for un-irradiated vehicle treated group (n = 2). NS, not statistically significant.

The two major causes of radiation-induced lethality is injury to the hematopoietic and gastrointestinal systems. We have previously shown that mice treated with either GT3 or recombinant TM were able to recover hematopoietic cells after TBI more efficiently compared to their respective vehicle treated groups. In the present study, we investigated the role of TM in hematopoietic cell recovery after irradiation. *TM* ^*Pro/-*^ and *TM* ^*+/+*^ mice were subjected to sub-lethal TBI (6 Gy) after pre-treatment with GT3 or vehicle for 24 h. Peripheral blood counts were obtained at 15 and 30 days. GT3 pre-treatment resulted in significant (*p < 0*.*05*) recovery of total WBC, neutrophils and lymphocytes at 30 day post-irradiation in *TM* ^*+/+*^ mice, but not in *TM* ^*Pro/-*^ mice, in comparison to their respective vehicle-treated irradiated groups (Fig 4). At day 15 no significant difference in terms of hematopoietic cell recovery was observed between *TM* ^*+/+*^ and *TM* ^*Pro/-*^mice as a result of GT3 treatment (data not shown). These data indicate that the post-TBI recovery of hematopoietic cells is more efficient in *TM* ^*+/+*^ mice than *TM* ^*Pro/-*^mice.

### Cytokine Induction in *TM* ^*+/+*^ and *TM* ^*Pro/-*^ Mice after GT3 Treatment

**Fig 5:**
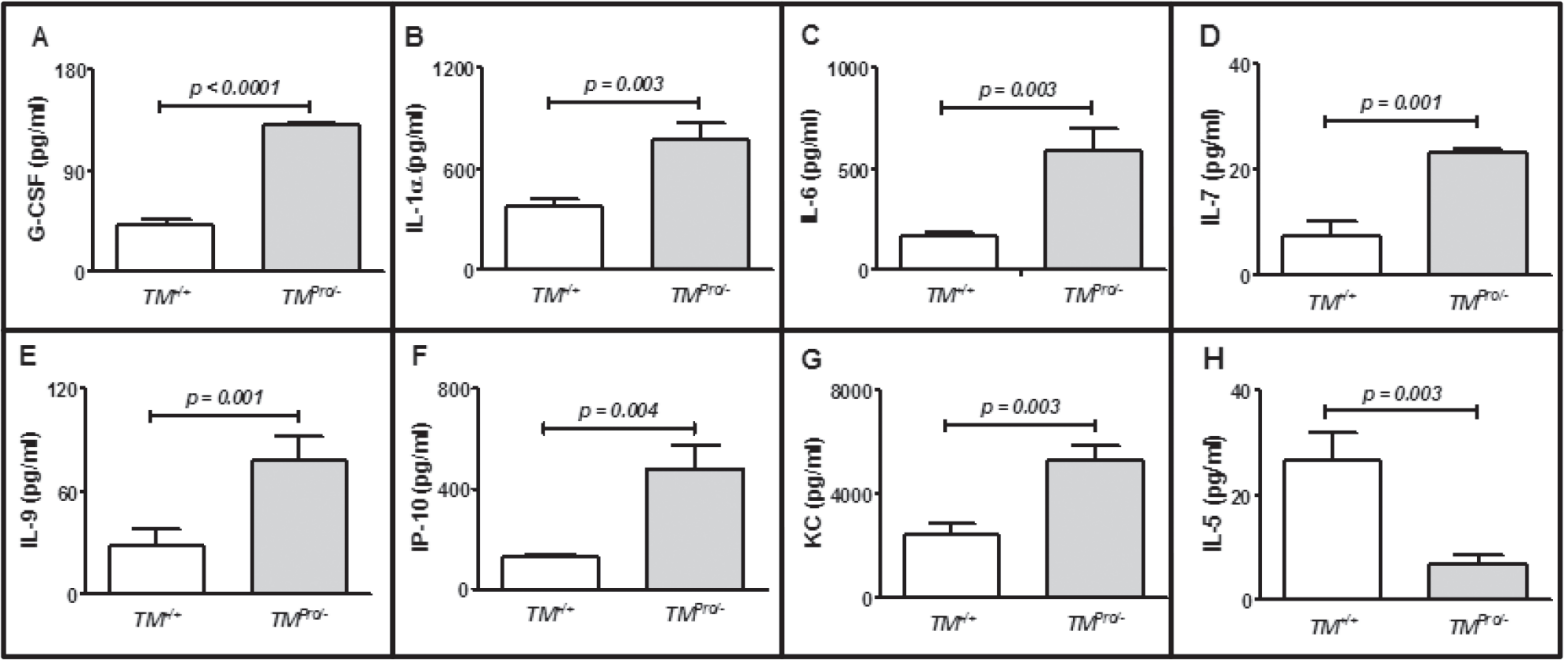
Cytokine- and chemokine expression in plasma of *TM* ^*+/+*^ and *TM* ^*Pro/-*^ mice 24 h after injection of GT3. Plasma G-CSF concentration was measured by mouse specific G-CSF ELISA kit after diluting plasma samples 100 folds. All other cytokines and chemokines were measured using multiplex Luminex on undiluted plasma samples obtained from *TM* ^*+/+*^ mice (n = 5) and age matched *TM* ^*Pro/-*^ mice (n = 4).

As G-CSF is significantly induced after GT3 treatment and plays a critical role in hematopoiesis, particularly in granulocytes generation and mobilization, we measured plasma G-CSF level in un-irradiated *TM* ^*+/+*^ and *TM* ^*Pro/-*^ mice after 24 h GT3 treatment. GT3 treatment significantly induced (*p < 0*.*001*) G-CSF in both *TM* ^*+/+*^ and *TM* ^*Pro/-*^ mice compared to their respective vehicle-treated groups (where plasma G-CSF concentration was below the detection limit). However, contrary to expectations, plasma G-CSF levels were about 3-fold higher in *TM* ^*Pro/-*^ mice than in *TM* ^*+/+*^ mice (Fig 5A). These data suggest that TM-dependent lethality protection by GT3 occurs by a G-CSF independent pathway.

We also assessed changes in the levels of other cytokines and chemokines that are directly or indirectly involved in WBC production or mobilization in plasma of *TM* ^*+/+*^ and *TM* ^*Pro/-*^ mice after 24 h of GT3 treatment. Significant increases (*p < 0*.*05*) in the levels of interleukin (IL)-1α, IL-6, IL-7, IL-9, interferon gamma-induced protein (IP)-10 and keratinocyte-derived chemokine (KC), the murine homolog of IL-8, were found in *TM* ^*Pro/-*^ mice compared to *TM* ^*+/+*^ mice (Fig 5B, 5C, 5D, 5E, 5F and 5G) after 24 h of GT3 treatment. Only the concentration of plasma IL-5 that plays a critical role in eosinophil mobilization from bone marrow to blood, was significantly elevated (*p = 0*.*01*) in *TM* ^*+/+*^ mice compared to *TM* ^*Pro/-*^ after 24 h GT3 treatment (Fig 5H).

## Discussion

TM is located on the luminal surface of most endothelial cells, where it forms a complex with circulating thrombin, thereby converting thrombin from a pro-coagulant to an anti-coagulant [14]. Thrombin, when in complex with TM, loses its ability to activate platelets and to convert fibrinogen to fibrin, and instead acquires the ability to activate protein C. APC has potent anti-coagulant, anti-inflammatory, and cyto-protective properties [15]. Deficiencies in endothelial TM are associated with a variety of disease states, including vascular diseases [16], graft-*versus*-host disease [17] and infectious diseases [18], and therapeutic use of recombinant TM or APC reduces mortality in patient with disseminated intravascular coagulation and/or severe sepsis [19–21]. Moreover, ionizing radiation reduces TM expression and function, both indirectly and directly [22, 23], and radiation-induced loss of TM appears to be involved in both early and delayed radiation toxicity [3, 24]. Conversely, systemic administration of recombinant TM or APC protects mice from lethality partly by accelerating recovery of hematopoietic cells [4].

Compared to α-tocopherol, GT3 regulates 8 times as many cellular genes [25] and has repeatedly been shown to be uniquely effective as a radioprotector [5, 6, 26]. Considerable circumstantial evidence and correlative data suggest that the superior radioprotective properties of GT3 are, at least partly, related to inhibition of HMGCR. However, while statins reduce the activity of HMGCR by competitive inhibition, GT3 acts by poly-ubiquitinating HMGCR, thus targeting the enzyme for proteasomal degradation [27]. Nevertheless, the end results with regard to HMGCR activity are similar, so it was reasonable to assume that GT3 would also enhance the expression of endothelial TM. The present study did confirm that ionizing radiation decreases the expression of endothelial TM, while GT3, similar to what is observed in response to statins albeit to a somewhat lesser degree, induced TM expression *in vitro*. More important, the experiments in the TM “deficient” (*TM* ^*Pro/-*^) and wild type (*TM* ^*+/+*^) mouse models demonstrated unequivocally that protection against radiation-induced lethality by GT3 is substantially reduced in the absence of TM. While TM, in addition to activating protein C, also activates thrombin-activatable fibrinolysis inhibitor (TAFI) and binds high mobility group box 1 (HMGB1) protein, the superior lethality protection observed in TM+/+ is likely related to generation of APC. Hence, we have shown that administration of wild-type APC as well as an anticoagulant variant of APC protects against TBI-induced lethality, while an anti-apoptotic variant of APC does not [4].

Multiple mechanisms contribute to TM deficiency in irradiated tissues. First, cytokines that are upregulated during the radiation response, for example, tumor necrosis factor α (TNFα), downregulate TM the transcriptional level [28]. Second, inflammatory mediators present during the early phase of radiation injury (eg, granulocyte elastase) cause shedding of TM from the endothelial cell membrane into the circulation [29]. Third, ionizing radiation directly inactivates TM by oxidation of a specific methionine (Met388) in the 6th epidermal growth factor-like domain [23]. Therefore, loss of functional endothelial TM in irradiated tissues is likely a result of combined downregulation at the gene level, ectodomain shedding, and oxidative inactivation. Because GT3 acts to increase TM gene expression, the effect is likely to be of benefit regardless of the extent of post-TBI ectodomain shedding and/or Met388 oxidation.

Previous studies have shown that GT3 provides considerable protection of hematopoietic cells in mice after exposure to ionizing radiation, mainly by mechanisms that appear to involve protection of hematopoietic stem and progenitor cells [26]. Accordingly, in the present study, post-TBI recovery of hematopoietic cells as a result of GT3 treatment was significantly better in *TM* ^*+/+*^ mice compared to *TM* ^*Pro/-*^ mice. The colony-stimulating factor, G-CSF, is generally assumed to play a central role in post-TBI hematopoietic recovery [30]. GT3 significantly induces G-CSF in mice [31], and a recent study showed that G-CSF neutralization abrogates GT3-mediated lethality protection in mice after exposure to TBI [32]. Interestingly and contrary to expectations, the current study showed an inverse relationship between G-CSF levels and neutrophil numbers and lethality protection, in that G-CSF levels 24 hours after GT3 injection were significantly higher in *TM* ^*Pro/-*^ mice than in *TM* ^*+/+*^ mice. These data suggest that enhancing G-CSF beyond a certain level does not contribute to post-TBI hematopoietic cell recovery and lethality protection. Our data are in agreement with those of other laboratories, showing that G-CSF deficient mice are capable of generating white blood cells in response to infection [33], thus clearly supporting the notion of G-CSF independent leukocyte production.

The present study also assessed the levels of various other cytokines and chemokines that are known to play critical role, directly or indirectly, in hematopoietic cell generation and mobilization. Similar to what was found for G-CSF, the levels of IL-1α, IL-7, and IL-9, all cytokines that enhance leukocyte formation, as well as of the leucocyte chemo-attractants, IP-10 and KC, were higher in *TM* ^*Pro/-*^ mice than in *TM* ^*+/+*^ mice after 24 h of GT3 treatment. On the other hand, the plasma levels of IL-6, which suppresses lymphopoiesis [34], were also elevated to a greater extent in *TM* ^*Pro/-*^ mice compared to *TM* ^*+/+*^ mice, while the levels of IL-5, which mobilizes leucocytes from bone marrow to blood [35], were significantly lower in GT3-treated *TM* ^*Pro/-*^ mice. Taken together it is tempting to speculate that the difference in lethality protection and hematopoietic recovery between the *TM* ^*+/+*^ mice and *TM* ^*Pro/-*^ mice may involve IL-5 and/or IL-6.

In conclusion our findings demonstrates that radiation causes decrease in the expression of endothelial TM, while GT3 induces TM; endothelial TM enhances post-TBI GT3-mediated lethality protection and hematopoietic cell recovery; and GT3-mediated lethality protection and hematopoietic cell recovery in irradiated mice is independent of G-CSF. These data may have implications to the basic understanding of the role of endothelial dysfunction in the radiation response and point to novel strategies to improve the efficacy of medical countermeasures used in radiological/nuclear emergency scenarios.
